# Challenges in diagnostic testing of nephritic factors

**DOI:** 10.3389/fimmu.2022.1036136

**Published:** 2022-11-14

**Authors:** Marloes A. H. M. Michels, Elena B. Volokhina, Nicole C. A. J. van de Kar, Lambertus P.W. J. van den Heuvel

**Affiliations:** ^1^ Department of Pediatric Nephrology, Amalia Children’s Hospital, Radboud University Medical Center, Nijmegen, Netherlands; ^2^ Innatoss Laboratories, Oss, Netherlands; ^3^ Department of Laboratory Medicine, Radboud University Medical Center, Nijmegen, Netherlands; ^4^ Department of Pediatrics/Pediatric Nephrology, University Hospitals Leuven, Leuven, Belgium; ^5^ Department of Development and Regeneration, University Hospitals Leuven, Leuven, Belgium

**Keywords:** nephritic factor, complement system, complement dysregulation, complement alternative pathway, assay standardization, C3 glomerulopathy, immune complex-mediated glomerulonephritis

## Abstract

Nephritic factors (NeFs) are autoantibodies promoting the activity of the central enzymes of the complement cascade, an important first line of defense of our innate immune system. NeFs stabilize the complement convertase complexes and prevent their natural and regulator-mediated decay. They are mostly associated with rare complement-mediated kidney disorders, in particular with C3 glomerulopathy and related diseases. Although these autoantibodies were already described more than 50 years ago, measuring NeFs for diagnostic purposes remains difficult, and this also complicates our understanding of their clinical associations. In this review, we address the multifactorial challenges of NeF diagnostics. We describe the diseases NeFs are associated with, the heterogenic mechanisms of action of different NeF types, the different methods available in laboratories used for their detection, and efforts for standardization. Finally, we discuss the importance of proper NeF diagnostics for understanding the clinical impact of these autoantibodies in disease pathophysiology and for considering future complement-directed therapy.

## 1 Introduction

**Figure 1:**
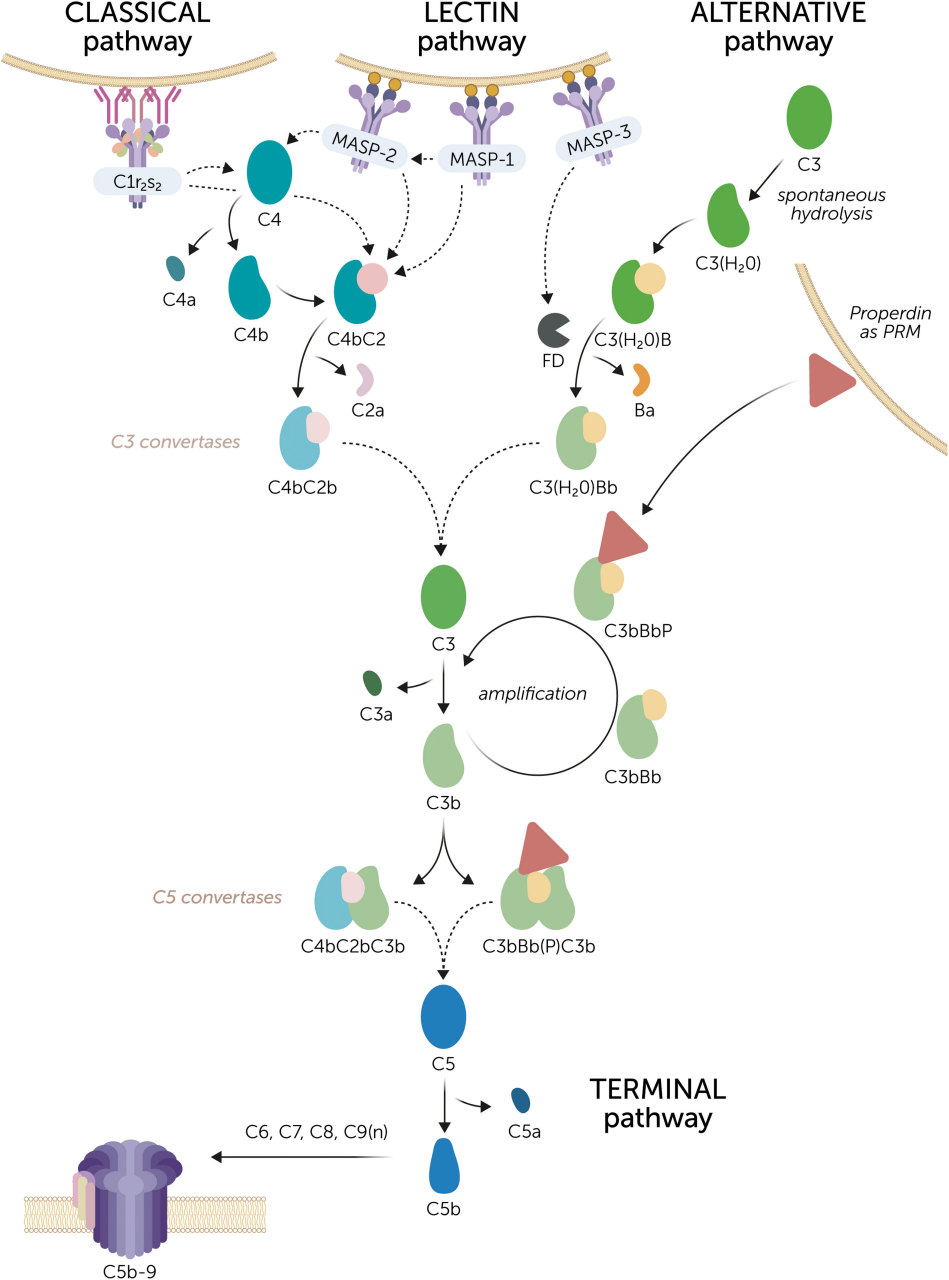
The complement system. Three routes can initiate complement activation. The classical pathway is activated by C1q coupled to the C1r2s2 proteases, together known as the C1 complex that recognizes, for example, antigen-antibody complexes. The lectin pathway is initiated by mannose-binding lectin (MBL), ficolins, or lectins, which recognize specific carbohydrate patterns on foreign surfaces. These pattern recognition molecules (PRMs) are coupled to the MBL-associated serine proteases (MASPs) MASP-1 or MASP-2. MASP-1 is autoactivated and is required to activate MASP-2. Activation of the classical and lectin pathway results in the cleavage of C4 and C2 by the C1r2s2 proteases and MASPs to form the C3 convertase C4bC2b. The alternative pathway is constantly active at a low rate due to the spontaneous hydrolysis of C3. Factor D (FD), which is activated by MASP-3, cleaves factor B to enable the formation of the initial C3 convertase C3(H_2_O)Bb. The C3 convertases convert C3 into C3a and C3b. Subsequently, the complement activation can be amplified *via* the alternative pathway. C3b can form new C3 convertase complexes that are stabilized by properdin (C3bBbP). Of note, properdin can also act as a PRM on certain surfaces to initiate alternative pathway activation. Upon continued C3 cleavage and C3b formation, C5 convertase complexes are formed, denoted as C4bC2bC3b and C3bBb(P)C3b, although their exact composition is unknown. C5 convertases cleave C5 into C5a and C5b, after which C5b recruits C6, C7, C8, and multiple copies of C9 to form the C5b-9 complex. This is the terminal complement complex that forms a pore and disrupts the membrane of the target cell. Dashed arrows indicate cleaving interactions.

With the enhanced understanding of the role of the complement system (**Figure 1**) in the pathogenesis of many diseases (1), the characterization and diagnosis of patients with complement-mediated disorders has gained a novel dimension. In-depth characterization of the complement dysregulation has become an important part of standard patient laboratory tests. More importantly, the acknowledgment of the role of complement has provided us with novel strategies to target complement-mediated diseases. After the success story of the complement inhibitor eculizumab in the treatment of atypical hemolytic uremic syndrome (2–4), great efforts have been made to achieve similar successes for the treatment of other diseases. Numerous new complement-directed therapeutics are currently in the drug development pipeline (5, 6). To keep up with the advances in drug development, it is crucial to have accurate and reliable assays available to characterize the complement activation status and complement-dysregulating factors in each patient.

One of the most challenging assays in the field of complement diagnostics is the detection of nephritic factors (NeFs). NeFs are stabilizing autoantibodies directed against the central enzymes of the complement system: the convertase complexes. By prolonging their activity and preventing their normal regulation, NeFs can contribute to an overactive complement system. Especially in the kidney disease C3 glomerulopathy (C3G), NeFs targeting the complement alternative pathway (AP) C3 convertase, i.e. C3NeFs, are considered the main driver of disease in many patients. However, robust and reliable standardized assays for C3NeF are lacking and this is hampering C3NeF research. According to the External Quality Assessment (EQA) rounds, organized by INSTAND e.V., the average success rate for C3NeF detection in the past five years (2016-2020) was 48% among the participating laboratories (7). This indicates that only half of all laboratories were able to correctly identify the presence or absence of C3NeFs in the reference samples sent around. Similar challenges apply for NeFs stabilizing the convertases of the complement classical pathway (CP), i.e. C4NeFs. Thus, important hurdles are still to be overcome in this field.

In this review, we provide an overview of the challenges in NeF testing and discuss the implications this has on our understanding of their role in disease. The two main factors complicating NeF analysis are the intrinsic heterogeneity of NeFs regarding their function and the large variety of methodologies used by different laboratories. Differences in NeF findings therefore hamper the research to their association with disease and/or disease activity. This underlines the need of standardization to aid research and diagnostics of NeFs and the diseases they are involved in.

## 2 C3 nephritic factor and C4 nephritic factor

**Figure 2:**
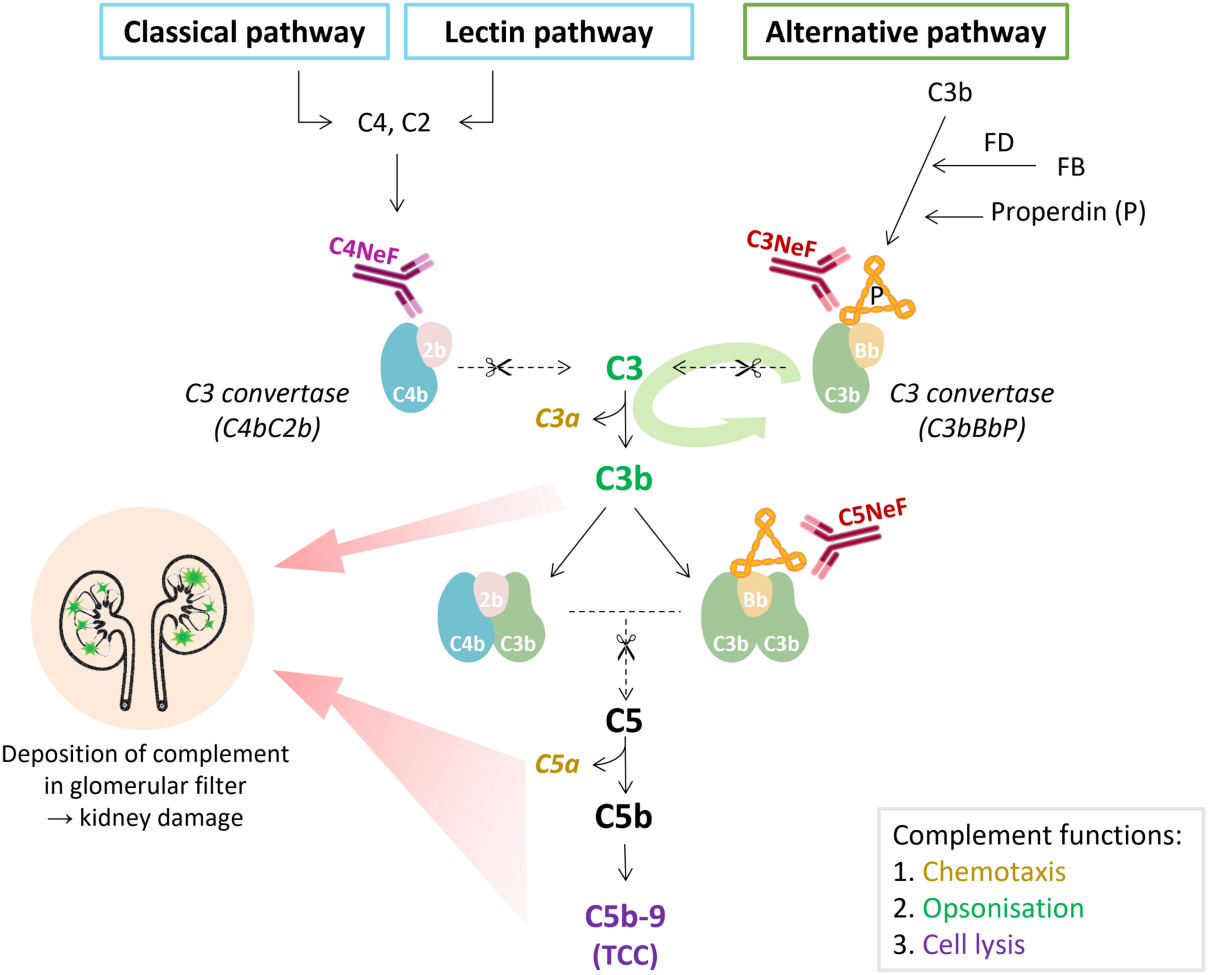
The place of nephritic factors in the complement system. C3 nephritic factors (C3NeF) stabilize the alternative pathway convertase complexes, whereas C4 nephritic factors (C4NeF) stabilize the convertases shared by the classical and lectin pathways. The term C5 nephritic factor (C5NeF) has been proposed for NeFs stabilizing alternative pathway C5 convertases. The green oval arrow illustrates the amplification loop of the alternative pathway. The colors of the complement components correspond to their function as indicated in the gray box. Overactivation of the complement system, mediated by NeFs, may result in the deposition of complement (breakdown) proteins in the glomerulus, inflammatory responses, and kidney injury. In C3 glomerulopathy, C3 degradation products are most abundant in the glomeruli, though terminal pathway proteins are also found. TCC, terminal complement complex.

In 1969, the term C3NeF was ascribed to a factor present in the serum of a hypocomplementemic membranoproliferative glomerulonephritis (MPGN) patient that specifically and very efficiently cleaved C3 when the serum was mixed with normal human serum (8). Later studies found that this C3NeF activity was mediated by stabilization of the AP convertase (9) and that C3NeFs had a heterogeneous immunoglobulin nature, mainly IgG (10–13). Therefore, nowadays, the term C3NeF is used to describe autoantibodies directed to neoepitopes of the AP C3 convertase that prolong the enzyme’s half-life, thereby resulting in enhanced cleavage of C3 (**Figure 2**).

Analogous to C3NeFs, in 1980 autoantibodies directed against the convertases shared by the CP and lectin pathway (LP) were reported (14, 15). These autoantibodies later became known as C4NeFs due to their capacity to stabilize the C4bC2b convertase complex (**Figure 2**). In many aspects, C4NeFs are functionally similar to C3NeFs, except for their specificity for convertases in another complement pathway. C4NeFs are IgGs recognizing neoepitopes in the CP/LP convertases to prolong their half-life and increase their ability to cleave C3.

## 3 Diseases associated with nephritic factors

C3NeFs are best described in the rare but severe kidney disease C3G. C3G is characterized by AP dysregulation leading to the deposition of C3 breakdown fragments in the glomeruli (16) (**Figure 2**). To establish the C3G diagnosis a kidney biopsy is required. The defining feature is a dominant staining for C3 visualized by immunofluorescence microscopy, i.e., a staining intensity of at least two orders of magnitude greater than staining for any other immune reactant (e.g., IgG, IgM, IgA, and C1q) (16–18). C3G can be subdivided into dense deposit disease (DDD) and C3 glomerulonephritis (C3GN) based on the glomerular complement deposition pattern visualized by electron microscopy. C3GN also encompasses CFHR5 nephropathy, which is a genetically driven familial form of C3G. The majority of the C3G patients show clear signs of AP activation in their blood. This includes increased markers of complement activation, e.g. C3 breakdown products and soluble (s)C5b-9, and low serum C3 levels, indicative of complement consumption due to excessive activation (19–22). C3NeFs are found in approximately 50% of the C3G cases, but the C3NeF prevalence among cohorts varies within a range of 26-75% (**Table 1**) (20–36). The large differences in these findings may not only be due to the different compositions of the patient cohorts, e.g. age of the patients and number of patients with DDD or C3GN, but also due to the heterogeneity of C3NeFs (see *section 4*) and the ability of the different assays used to detect all C3NeFs (see *section 5*).

**Table 1:** Prevalence of C3 nephritic factor (C3NeF) in C3 glomerulopathy (C3G) cohorts.

Study	Year	Country	Total C3G cohort (n)	Pediatric onset^a^	Tested for C3NeF (n)	C3G	DDD	C3GN
				(% of total)		(% C3NeF)	(% C3NeF)	(% C3NeF)
**Nasr et al.** (23)	2009	USA	32	44%^b^	9		78%	
**Sethi et al.** (24)	2012	USA	12	17%^c^	10			50%
**Zhang et al.** (25)	2012	USA	32	71%^c^	32		78%	
**Servais et al.** (26)	2012	France	85	36%^b^	75	57%	86%	45%
**Rabasco et al.** (27)	2015	Spain	60	n.r.	23			48%
**Corvillo et al.** (28)	2016	Spain	80	n.r.	49	35%	70%	10%
**Iatropoulos et al.** ^d^ (29)	2016	Italy	73	n.r.	n.r.	54%	78%	44%
**Zhang et al.** (30)	2017	USA	168	n.r.	168	52%	72%	38%
**Marinozzi et al.** (20)	2017	France	127	45%^c^	101	75%		
**Bomback et al.** (31)	2018	USA	111	32%^c^	51	27%	11%	31%
**Ravindran et al.** (32)	2018	USA	114	n.r.	69	43%	30%	46%
**Iatropoulos et al.** ^d^ (22)	2018	Italy	93	68%^c^	85	49%	78%	38%
**Michels et al.** (33)	2018	NL	27	n.r.	27	59%	67%	57%
**Garam et al.** (34)	2020	Europe^e^	48	n.r.	47	26%	45%	19%
**Levine et al.** (35)	2020	UK	61	n.r.	41	44%	59%	33%
**Wong et al.** (36)	2021	UK	39	100%	36	39%	62%	26%
**Michels et al.** (21)	2022	NL	29	100%	28	71%	67%	80%

USA, United States of America; NL, The Netherlands; UK, United Kingdom; DDD, dense deposit disease; C3GN, C3 glomerulonephritis; n.r., not reported.

a Age at time of investigation may be different in some studies.

b <16 years at onset.

c <18 years at onset.

d Additional collaborations with centers in Israel, Portugal, France, Switzerland, Russia, and Turkey.

e 34 centers in Central and Eastern Europe.

C3NeFs are also found in a substantial number of patients with idiopathic (primary) immune complex-mediated MPGN (IC-MPGN). The prevalence in patient cohorts is reported between 23% and 54% (22, 26, 29, 34–36). The pattern of glomerular injury in IC-MPGN resembles the injury in C3G, but IC-MPGN is distinguished from C3G by the substantial presence of immunoglobulin deposits, which can cause CP activation in the glomerulus. However, the finding that genetic and acquired abnormalities of the AP, including C3NeFs, are found in both diseases suggests they may share a disease spectrum (37). This is also supported by cases in which the IC-MPGN pattern evolves to a C3G pattern in subsequent biopsies or vice versa.

Next to these glomerular kidney diseases, C3NeFs are common in patients with acquired partial lipodystrophy (APL). APL is an extremely rare disorder characterized by fat loss in the upper half of the body, usually occurring during childhood or adolescence (38). Most patients have C3 hypocomplementemia and in approximately 70-80% of the cases C3NeFs are found, especially in those with low serum C3 levels (38–40). Supposedly as a result of the C3NeF presence, approximately 20% of the patients develop C3G (38, 41, 42).

Lastly, C3NeFs have been described, although much less frequently, in patients with meningococcal infections and disease (43–45), in patients with systemic lupus erythematosus (SLE), who often also showed presence of APL and/or C3G (46–48), and in post-infectious glomerulonephritis (49–51).

C4NeFs have been reported in several diseases as well. The first reports from 1980 identified C4NeFs in a patient with acute post-infectious glomerulonephritis (15) and in patients with SLE (14). Later studies have described C4NeFs in more patients with SLE (52, 53), in patients with MPGN (52, 54, 55), in patients with Sjögren syndrome (52), and in a patient with sepsis caused by an *N. meningitidis* infection (56). More recently, the focus of C4NeF research has been on C3G and IC-MPGN. C4NeFs have been reported in two C3G cohorts with an incidence of 3% (30) and 8% (57) and in mixed C3G/IC-MPGN cohorts with an incidence of 6% (58) and 14% (59). Interestingly, in a substantial proportion of these patients both C4NeFs and C3NeFs are found (30, 52, 54, 55, 58, 59).

## 4 Nephritic factor heterogeneity

C3NeFs form a heterogeneous group of immunoglobulins that likely bind to different epitopes of the AP convertase. First, they differ in the extent to which they prolong the half-life of the C3 convertase (20, 25, 60), ranging from tens of minutes to even hours. Second, C3NeFs confer the convertase with varying resistance against the various complement regulatory proteins. C3NeFs may interfere with Factor H (FH) (60–65), complement receptor 1 (CR1) (60, 66), and decay-accelerating factor (DAF) (60, 67), although the efficiency (for each regulator) differs between NeFs of different patients. Few C3NeFs were found that did not or hardly interfere with any of these regulators of extrinsic decay (60). Third, C3NeFs differ in their dependence on the convertase-stabilizing protein properdin. Some C3NeFs are only active in the presence of properdin, i.e. properdin-dependent NeFs, whereas other C3NeFs also, or only (20), act on convertases without properdin, i.e. properdin-independent NeFs (20, 25, 60, 62, 68–70). Finally, it has been shown that NeFs can differ in their ability to affect C5 convertase activity and C5 conversion, sometimes referred to as C5NeF activity (20, 60–62, 71) (**Figure 2**). It is this marked heterogeneity that has complicated our understanding of C3NeFs since their discovery until today.

Although less studied, C4NeFs also form a heterogeneous group. Convertase stabilization through interference with complement regulator-mediated extrinsic decay has been described for C4 binding protein (C4BP) (30, 56, 72, 73), CR1 (30, 56, 74), and DAF (57, 67). Some studies have shown the specific ability of C4NeFs to also stabilize the C5 convertase complex C4bC2bC3b (56, 73), suggesting also C4NeFs may recognize different epitopes in different patients. However, it is important to note that research on the stabilization of CP/LP and especially AP C5 convertases by NeFs is hampered by the unknown exact structure of the C5 convertase complex and the transient nature between convertase complexes that cleave C3 and C5.

## 5 Nephritic factor detection methods

**Figure 3:**
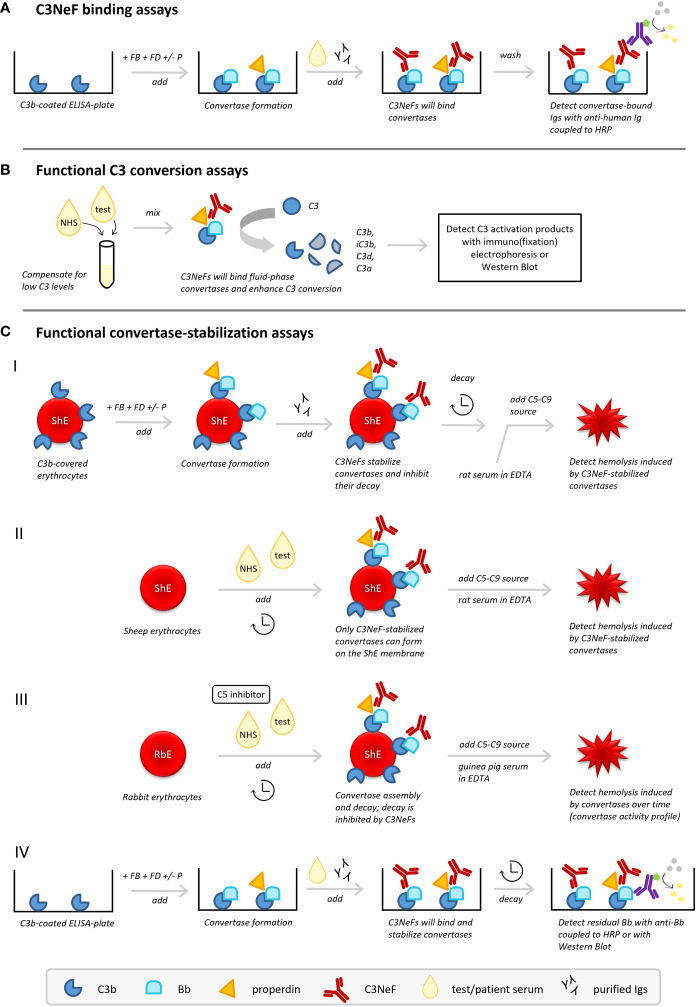
C3 nephritic factor (C3NeF) detection methods. Three types of methods to detect C3NeFs can be distinguished: **(A)** assays detecting NeF binding to the convertase, **(B)** functional assays measuring C3 conversion influenced by NeFs, and **(C)** functional assays measuring convertase stabilization by NeFs. ELISA, enzyme-linked immunosorbent assay; FB, Factor B; FD, Factor D; HRP, horseradish peroxidase; Igs, immunoglobulins; NHS, normal human serum; P, properdin; RbE, rabbit erythrocytes; ShE, sheep erythrocytes.

A major bottleneck in NeF research, related to the heterogeneous nature of NeFs, is the difficulty and diversity of detection methods, which are often only available in specialized laboratories. Many different assays are being used for NeF identification and tests differ in sensitivity and specificity. C4NeF testing is available in even fewer laboratories than C3NeF testing, so here we focus on the methods described for C3NeF detection, which often have a parallel version for detecting C4NeFs. NeF assays can generally be divided into three groups (**Figure 3**): [1] binding assays that detect the binding of the NeF to the convertase complex, [2] functional assays that measure the C3 activation products, and [3] functional assays that detect the stabilization of the convertase. C3NeFs can be variably positive in these different assays, hence some laboratories use a combination of them for C3NeF detection (25, 60).

### 5.1 C3NeF binding assays

C3NeF binding assays (**Figure 3A**) are usually enzyme-linked immunosorbent assay (ELISA)-based methods aimed to detect the interaction of the C3NeF antibody with its target, i.e. plate-bound AP convertases formed from purified complement components (25, 60, 61, 75). Typically, plates are coated with C3b; then Factor B (FB) and Factor D (FD), in presence or absence of properdin, are added to allow formation of AP convertase complexes. After washing, or simultaneous with the addition of purified AP components, the test sample is added, i.e. purified patient immunoglobulins or patient sera. If C3NeFs are present, their binding can be detected *via* horseradish peroxidase-conjugated anti-human immunoglobulin antibodies supporting substrate conversion. Importantly, a C3b-only well should be used as a blank control in these methods.

In general, binding assays are easy to implement in laboratories and directly identify the immunoglobulin nature of the factor interacting with the enzyme complex. However, these assays are not functional assays, so they may detect a binding interaction that might not influence enzyme function. Besides this chance on false-positive results, the assay may also give false-negative results, as the convertases formed on microtiter plates can adopt artificial conformations that may not be recognized by all NeFs (**Table 2**).

**Table 2:** Advantages and limitations of the different nephritic factor (NeF) detection assays.

Assay type	Advantages	Limitations
**C3NeF binding assays**	• Easy to implement (ELISA) • Directly identify immunoglobulin nature	• Detect binding, not function • Artificial conformations of convertases possible
**Functional C3NeF assays measuring C3 cleavage**	• Detect a functional effect	• Non-specific^a^
**Functional C3NeF assays measuring convertase stabilization**	• Detect a functional effect	
*** ShE with purified complement components***	*• Physiological conformations of convertases on a membrane* *• Directly identify immunoglobulin nature*	*• Require expertise^b^* *• Time-consuming*
*** ShE with whole serum***	*• Physiological conformations of convertases on a membrane* *• Physiological conditions of convertase assembly and decay*	*• Require expertise^b^* *• Lysis may occur in step 1*
*** RbE with whole serum***	*• Physiological conformations of convertases on a membrane* *• Physiological conditions of convertase assembly and decay*	*• Require expertise^b^* *• Additional approaches required to identify immunoglobulin nature*
*** ELISA-based***	*• Easy to implement (ELISA)*	*• Artificial conformations of convertases possible*

ELISA, enzyme-linked immunosorbent assay; ShE, sheep erythrocytes; RbE, rabbit erythrocytes.

a The assay described by Zhao et al. (61) has increased specificity compared to the original assays.

b Hemolytic assays are dependent on the availability of (fresh) erythrocytes and are prone to batch-to-batch variability of erythrocytes.

### 5.2 Functional C3NeF assays measuring C3 activation products

Functional assays measuring the activation products of C3 (**Figure 3B**) were the first assays described for measuring C3NeF activity (8, 76). These tests measure the release of C3 breakdown products after mixing the test sample with control serum by immunoelectrophoresis or western blotting (25, 60). Many patients with C3G and other diseases related to C3NeF have low serum levels of C3 as a result of consumption. Hence, mixing with control serum to supply uncleaved C3 is essential to allow formation of fluid phase AP convertases to be stabilized by the NeFs from the test sample. C3NeF positivity will then result in increased C3 conversion.

The advantage of these assays is that they are detecting a functional enzyme effect. However, increased C3 breakdown may also be caused by other factors, so a positive outcome does not have to be specific for presence of C3NeFs (**Table 2**).

Zhao et al. recently described a variant on this assay. Fluid phase C3 convertases were assembled with purified components, which were then mixed with patient immunoglobulins and additional C3 (61). C3 conversion was measured by the release of C3a detected by ELISA.

### 5.3 Functional C3NeF assays measuring convertase stabilization

Functional assays measuring the convertase-stabilizing function of C3NeFs were developed later, mostly in the form of hemolytic assays (9) (**Figure 3C**). These assays have supplemented or replaced many of the previous assays and are frequently used today. Although there are variations in the exact procedures, AP hemolytic C3NeF assays generally follow the same principle: to measure the activity of C3NeF-stabilized convertases on the erythrocyte membrane *via* C5b-9-induced hemolysis. Hemolysis provides the easy readout of released hemoglobin that can be detected spectrophotometrically.

#### 5.3.1 Sheep erythrocyte hemolytic assays with convertases formed from purified components

In the first variant, sheep erythrocytes are used as the platform for formation of AP convertases with purified complement components (**Figure 3C.I**). Since sheep erythrocytes are rich in sialic acids and therefore naturally protected against human complement attack by recruiting the major AP regulator FH (77, 78), specific approaches need to be applied to cover the sheep erythrocytes with initial C3b. C3b can be deposited on the sensitized, i.e. anti-sheep antibody-treated, sheep erythrocyte membrane *via* CP convertase intermediates (79, 80) or on unsensitized sheep erythrocytes by using FB-partially inactivated/FH-depleted serum (25). Subsequently, AP convertases are built from purified components (FB and FD, with or without properdin) followed by the addition of patient immunoglobulin fractions as a potential source of C3NeFs. Importantly, convertases are then left to decay for a set time period. After this incubation, the residual activity of convertases is examined by the addition of rat serum diluted in ethylenediaminetetraacetic acid (EDTA) as a heterologous source of C5-C9. This allows stabilized convertases to cleave C5 and induce C5b-9-mediated hemolysis. Rat serum, as well as guinea pig serum, is compatible with human complement and more potent in generating C5b-9; the EDTA prevents *de novo* convertase formation from these sera. If no C3NeFs are present in the test sample, the convertases are broken down after a certain decay time and no hemolysis is observed. However, if hemolysis is observed, it indicates that convertases have been stabilized by C3NeFs present in the test sample, allowing them to induce C5b-9 formation (20, 25, 81).

#### 5.3.2 Sheep erythrocyte hemolytic assays with convertases formed from whole serum

Another widely applied hemolytic C3NeF assay combines unsensitized sheep erythrocytes with a mix of control serum and test serum (82–84) (**Figure 3C.II**). This method exploits the ability of C3NeFs to form and stabilize convertase complexes on the surface of the naturally non-activating sheep erythrocyte by overcoming the negative regulation; an action that cannot be fulfilled by serum in the absence of C3NeFs (83). A limited time frame is used to restrict the complement activation by the test sample to convertase assembly and to prevent lysis. Hemolysis is only induced in a standardized second step by addition of rat serum in EDTA-buffer. If no hemolysis is seen, no C3NeFs were present and no convertases could be formed on the sheep erythrocyte membrane. Although this time-dependent separation of convertase assembly and hemolysis will apply for most samples, it should be noted that some patient samples *are* able to cause (unwanted) hemolysis in the convertase formation step of those assays (85) (**Table 2**).

#### 5.3.3 Rabbit erythrocyte hemolytic assays with convertases formed from whole serum

Recently, an adapted variant of this assay using rabbit erythrocytes (86, 87) was optimized for C3NeF diagnostics (33) (**Figure 3C.III**). Rabbit erythrocytes have a low sialic acid content and are therefore potent AP activators. When mixed with control serum and test serum, AP convertases will spontaneously assemble on their membrane. Importantly, a C5 inhibitor, e.g. eculizumab, is added here to strictly prevent hemolysis during this first step and to separate convertase assembly from hemolysis readout. After washing of the erythrocytes, guinea pig serum in EDTA is added to allow preformed convertases to cleave C5 and induce C5b-9-mediated hemolysis. In contrast to previous assays, the full convertase activity profile with convertase assembly and decay is visualized by using different incubation periods for the test serum incubation. Compared to control serum showing a profile with convertase assembly and clear convertase breakdown, serum containing C3NeFs will show a prolonged convertase activity profile with persistently high levels of lysis over time, indicative of stabilized convertases.

Of note, both this assay and the assay described in *section 5.3.2* do not directly identify C3NeFs, since the immunoglobulin nature of the convertase-stabilizing factor in serum is not confirmed. Mixing control serum with patient immunoglobulin fractions as an extra test overcomes this limitation and will specify if the convertase-stabilizing factor is an autoantibody (33). On the other hand, assessing the kinetics of convertase assembly and decay in complete serum offers the advantage of the physiological environment. All complement regulators and other serum factors that may influence convertase activity are present, so this maximizes the chance of detecting functionally relevant pathogenic factors (**Table 2**).

#### 5.3.4 ELISA-based convertase stabilization assays

The principles of convertase stabilization by NeFs in the hemolytic assays above, especially the one described in *section 5.3.1*, have also been translated to assays analyzing the kinetics of convertase dissociation on a microtiter plate (**Figure 3C.IV**). AP convertases are built from purified components on a C3b-coated microtiter plate after which serum or patient immunoglobulin fractions are added. Instead of hemolysis, residual plate-bound Bb fragments detected after a certain time of decay are used as a measure of intact convertases stabilized by C3NeFs (60, 61). Recently, also a variant of this ELISA-based assay was described that combined convertase formation and decay on microtiter plates with residual Bb analysis on western blot (62).

Of note, these assays do not assess the C3/C5 cleaving capacities of the C3NeF-stabilized convertases. Furthermore, in hemolytic assays, convertases are formed on a membrane surface in their natural orientation *via* the reactive thioester of C3, whereas convertases formed on microtiter plates may adopt artificial configurations. Nonetheless, the availability of erythrocytes and batch-to-batch variability are a major challenge of hemolytic assays, making these assays often only available in specialized labs. In contrast, ELISA techniques are much more easy to implement (**Table 2**).

## 6 Efforts for assay standardization

As stated before, C3NeF detection in general is problematic, with the EQA rounds from 2016-2020 reporting success rates for identifying the reference samples between 31% (2020) and 75% (2016) (7). Thus, if results of a sample are compared between laboratories, or even between different tests within one laboratory, variable outcomes are obtained. At the moment, a golden standard is lacking and there is a high need for standardization. The same holds true for C4NeF assays.

First, it is important to note that properdin-dependent C3NeFs are missed in assays that study the stability of C3bBb convertases formed from purified components without properdin. In line with this, previous reports describing panels with multiple C3NeF assays have shown that additional NeFs could be identified when properdin was incorporated in the test settings (20, 25, 60, 62). Marinozzi et al. also identified a group of C3NeFs that was only detected in absence of properdin (20), and this is supported by our own unpublished observations. However, it remains to be investigated what the physiological relevance of these NeFs is, as in physiological conditions properdin is always present in the circulation.

Recently, a *CFB* construct based on the gain-of-function variant p.Lys323Glu was proposed as a standardized positive control for C3NeF assays (88). This genetic variation is associated with atypical hemolytic uremic syndrome and has previously been shown to result in a stabilized convertase complex that is more resistant to negative regulation (89). The construct caused prolonged convertase activity reminiscent of that of patients with C3NeF activity (33, 88). Therefore, this might be a good alternative for patient serum or patient immunoglobulins, of which material is limited. For C4NeF assays, a similar approach could be followed, as recently also *C2* gain-of-function variants resulting in stabilized CP convertase complexes were identified (90).

Furthermore, surfaces other than erythrocytes have been described for convertase analysis, namely magnetic beads and liposomes (91, 92). These platforms may be more suitable for standardization due to lower batch-to-batch variability. However, these setups have not yet been validated for C3NeF or C4NeF detection. Besides, magnetic beads are a less physiological model than erythrocytes. The optimal platform for the detection of functionally relevant NeFs should still be determined. Thus, future research may not only focus on improved standardization but also on further assay optimization with different (cellular) targets for convertase assembly.

## 7 Clinical implications

Assay standardization may also lead to a better understanding of the role of C3NeFs and C4NeFs in disease. For a long time, it has been difficult to relate C3NeF presence with complement (activity) markers and clinical parameters, such as disease activity and outcome. Nonetheless, increased interest in this field of research has led to major progresses during the last decade. Here, we will discuss some of the main findings of the clinical associations of NeFs, with a focus on C3G/IC-MPGN.

### 7.1 Clinical associations of C3NeFs

By stabilizing AP convertases, C3NeFs increase the potential of C3 conversion, which may lead to consumption of C3. Indeed, previous studies have associated the lowered C3 levels in patients with C3G with the presence of C3NeFs (20, 26, 29, 93). Recently, two studies on pediatric C3G cohorts showed that although C3 levels at presentation did not differ between patients with or without C3NeFs, the last measured C3 levels during follow-up were significantly lower in the patients that had tested positive for C3NeF (21, 36). This indicates the presence of C3NeF was associated with a higher likelihood of continued C3 consumption. In line with this, a previous report showed permanently low C3 during follow-up in 67% (12/18) of the C3NeF-positive children with C3G (94).

In a subset of patients with C3G, the complement system is dysregulated up to the level of the terminal pathway as seen by elevated sC5b-9 levels. This implies that there is also enhanced activation of the C5 convertases in those patients, and it was already hypothesized in 1986 that different NeF types may lie at the basis of this difference (71). Several studies have related properdin-dependent C3NeFs to (stronger) terminal pathway activation and to the C3GN phenotype (20, 62, 68, 69). On itself, C3GN is also associated with more pronounced terminal pathway activation (e.g. higher sC5b-9 and lower C5 and properdin) compared to the DDD subtype (19, 21). In 2017, the term C5 nephritic factor (C5NeF) was coined for NeFs that stabilize the AP C5 convertase. This was based on the finding that these NeFs stabilized convertases formed from purified proteins with properdin (C3bBbP) and were correlated with increased sC5b-9 levels (20). Some patients with C5NeFs were also C3NeF positive, i.e. they also tested positive when the stability of convertases formed without properdin (C3bBb) was assayed. In this regard, single C5NeF positive patients as described by this study correspond to the patients with properdin-dependent C3NeFs, and C3NeF/C5NeF double-positive patients correspond to patients with properdin-independent C3NeFs. Importantly, the exact composition and conformation of AP C5 convertases (and CP/LP C5 convertases) remain unknown. Furthermore, some patients with presumed C5NeFs (isolated or in combination with C3NeFs) do not show C5 convertase dysregulation, i.e. increased sC5b-9 levels, and conversely, some patients without C5NeFs do have increased sC5b-9 levels (20, 62). Moreover, complement markers are not always available. Therefore, the application of C5NeF terminology may be confusing, as it is unclear whether the C5NeF terminology is directly interchangeable with the properdin-dependency of NeFs and whether it may be used regardless of C5 convertase dysregulation. This is why it may be preferable to classify the NeF types based on their dependence on properdin for recognizing the convertase and to refrain from implications on the specific stabilization of the C5 convertase complex.

Even though C3NeFs are the most commonly found AP aberrations in C3G, not much is known about their behavior over time in the course of the disease and their relation to clinical outcome. While some patients have been shown to maintain their C3NeF activity for more than 10 years (58), C3NeF findings may also fluctuate over time, with some patients eventually losing their C3NeFs (26, 33, 93, 94). The longitudinal follow-up of C3NeFs is often not part of standard patient investigations. Moreover, many studies did not find correlations between C3NeF activity and clinical parameters (93, 94), although some studies have shown that patients with C3NeFs were more likely to have a better outcome (20, 29).

### 7.2 Clinical associations of C4NeFs

Lately, C4NeF research has also mostly focused on C3G and IC-MPGN. One of the most important questions here is how and to what extent a CP-dysregulating factor can contribute to AP-driven disease. In many patients with C3G, the disease is preceded by an infectious trigger, which exposes C4NeF epitopes. C3G shows many clinical similarities with post-infectious glomerulonephritis, such as low serum C3 levels, but in contrast to C3G, the C3 consumption and disease generally resolve within weeks or months (95, 96). However, some (atypical) cases evolve to C3G, indicating that an initial CP-mediated response may evolve to a primarily AP-driven pathology, likely *via* the AP amplification loop. Possibly, C4NeFs elicit such a similar mechanism in C3G and IC-MPGN.

The majority of C3G/IC-MPGN patients with C4NeFs are characterized by strong complement activation up to the level of the terminal pathway, especially patients positive for both C4NeFs and C3NeFs (30, 54, 58, 59). This double positivity for both C4NeF and C3NeF occurs in many cases and makes it difficult to determine the individual contribution of both NeFs to the disease. Furthermore, C4NeFs may persist over the disease course and during partial remission up to 70 months (58).

Studies on the relationship between C4NeF activity and clinical markers are scarce and often lack power due to the low number of positive patients. Besides, the scarce literature shows conflicting results. In a cohort with 100 hypocomplementemic MPGN patients described in 1994, nephrotic syndrome and poor prognosis occurred more frequently in C3NeF/C4NeF double-positive patients compared to single C3NeF or single C4NeF-positive patients (54). A recent study with 119 IC-MPGN/C3G patients showed that C4NeF-positive and C3NeF/C4NeF double-positive patients less frequently had a lowered eGFR at presentation and that, during the follow-up, none of the 17 patients reached kidney failure requiring kidney replacement therapy (in contrast to 17/92 C4NeF-negative patients) (59). In a study of our group, we did not observe an abnormal clinical presentation or disease course compared to C3G/IC-MPGN patients without C4NeFs (58).

## 8 Conclusions and future directions

With the increase in complement therapeutics, laboratory analysis of the complement system has become increasingly important (97). For example, it can be key in monitoring complement-directed therapy, as has been shown for the C5 inhibitor eculizumab in atypical hemolytic uremic syndrome (98, 99). It is well-known that accurate NeF analysis is challenging. This is due to the heterogenic nature of NeF autoantibodies, including differences in properdin-dependency, and due to inconsistent outcomes of samples tested in different assays between laboratories or even between different assays within one laboratory. As outlined in this review, each assay has its own advantages and disadvantages. There is a high need for comparison of NeF findings between groups. The considerable inconsistency between cohort findings now complicates the interpretation of the results of the NeF associations with complement biomarkers and/or disease parameters. Assay standardization, in combination with longitudinal analysis of NeFs and all relevant complement and clinical parameters in patients, may help to reveal different pathophysiological mechanisms of complement dysregulation in different patient groups. It may even provide more insight in disease outcome. Iatropoulos et al. showed that disease outcome was better predicted when patients were divided in groups based on cluster analysis, including not only pathology findings but also complement (e.g. NeF presence) and clinical findings, than based on the traditional subdivision into C3GN, DDD, and IC-MPGN (22). Moreover, our group showed *in vitro* proof that properdin inhibition may be a potential novel therapeutic approach for patients with properdin-dependent NeFs, likely regardless of disease classification (70). Thus, accurate and reliable NeF diagnostics are essential to better understand disease, to characterize patient (sub)groups and to move forward towards more specific complement-directed therapy in different patient groups.

## Author contributions

MM wrote the manuscript. EV, NK, and LH critically reviewed the manuscript. All authors listed have made a substantial, direct and intellectual contribution to the work, and approved it for publication.

## Funding

This work was supported by the Dutch Kidney Foundation (13OCA27 COMBAT Consortium and 20OK021 PERSPECTIV).

## Acknowledgments

This study was performed on behalf of the COMBAT Consortium. This is an interuniversity collaboration in the Netherlands that is formed to study basic mechanisms, assay development, and therapeutic translation of complement-mediated renal diseases. Principal investigators are (in alphabetical order): S. Berger (Department of Internal Medicine-Nephrology, University Medical Center Groningen, Groningen, Netherlands), J. van den Born (Department of Internal Medicine-Nephrology, University Medical Center Groningen, Groningen, Netherlands), P. Gros (Department of Chemistry, Utrecht University, Utrecht, Netherlands), L. van den Heuvel (Department of Pediatric Nephrology, Radboud University Medical Center, Nijmegen, Netherlands), N. van de Kar (Department of Pediatric Nephrology, Radboud University Medical Center, Nijmegen, Netherlands), C. van Kooten (Department of Internal Medicine-Nephrology, Leiden University Medical Center, Leiden, Netherlands), M. Seelen (Department of Internal Medicine-Nephrology, University Medical Center Groningen, Groningen, Netherlands), A. de Vries (Department of Internal Medicine-Nephrology, Leiden University Medical Center, Leiden, Netherlands). NK and BH and are members of the European Reference Network for Rare Kidney Diseases (ERKNet)-Project No 739532.

## Conflict of interest

The authors declare that the research was conducted in the absence of any commercial or financial relationships that could be construed as a potential conflict of interest.

## Publisher’s note

All claims expressed in this article are solely those of the authors and do not necessarily represent those of their affiliated organizations, or those of the publisher, the editors and the reviewers. Any product that may be evaluated in this article, or claim that may be made by its manufacturer, is not guaranteed or endorsed by the publisher.
